# Longitudinal changes in life-space mobility and autonomy in participation outdoors among Finnish community-dwelling older adults from pre-COVID-19 to through the pandemic

**DOI:** 10.1007/s40520-024-02734-6

**Published:** 2024-04-01

**Authors:** Katja Lindeman, Laura Karavirta, Kaisa Koivunen, Kirsi E. Keskinen, Johanna Eronen, Erja Portegijs, Taina Rantanen

**Affiliations:** 1 Faculty of Sport and Health Sciences and Gerontology Research Center, University of Jyväskylä, Jyväskylä, Finland; 2 Center of Human Movement Sciences, University of Groningen, University Medical Center Groningen, Groningen, The Netherlands

**Keywords:** Ageing, COVID-19, Driving, Mobility, Physical performance

## Abstract

**Background:**

Among older people, community mobility was reduced at the beginning of the COVID-19 pandemic, but the longer-term changes are unclear.

**Aims:**

To study lower extremity performance and car driving as predictors of changes in older adults’ life-space mobility, autonomy in participation outdoors, and the risk of developing restricted life-space mobility from 2017 to 2022.

**Methods:**

Life-space mobility (scoring range 0-120) and autonomy in participation outdoors (scoring range 0–20) were assessed in community-dwelling individuals (*n* = 657) in 2017–2018 (baseline age 75, 80, or 85 years), during the first wave of COVID-19 in 2020, and in 2021–2022. Lower extremity performance was assessed using the Short Physical Performance Battery, and car driving was self-reported at baseline. Data were analysed using generalized estimating equations and Cox regression.

**Results:**

During the first wave of COVID-19 in 2020, life-space mobility decreased on average by 10.3 (SD 21.6) points and partially recovered in 2021–2022 (+ 2.7, SD 21.8). The same pattern was observed for autonomy in participation outdoors. Non-drivers and those with impaired lower extremity performance had a 2.4-to-3.6-fold adjusted risk of developing restricted life-space mobility over the follow-up period compared to drivers with intact lower extremity performance.

**Conclusions:**

For older people, the recovery of community mobility was incomplete after the restrictions stemming from the pandemic were lifted. Older adults with impaired lower extremity performance and who did not drive were particularly vulnerable to developing restricted life-space mobility, a situation that could lead to social isolation and reduced well-being.

**Supplementary Information:**

The online version contains supplementary material available at 10.1007/s40520-024-02734-6.

## Introduction

In March 2020, the World Health Organization declared COVID-19 a global pandemic. Since then, measures to reduce spread and transmission of the virus, particularly in the early stages of the pandemic, hindered older adults’ possibilities to carry out daily errands and participate in activities outside their homes [1]. Reduced mobility and limited participation in the community can be hazardous to older people, as the freedom to come and go is essential for active and healthy ageing [2]. Therefore, the COVID-19 pandemic and restrictions may have had a far-reaching impact on older adults, even though the restrictions have now been lifted.

Life-space mobility, i.e., the frequency and area that a person moves in a given period, conveys an active approach to life and access to community facilities outside the home. The University of Alabama at Birmingham Study of Aging Life-Space Assessment (UAB-LSA) is a commonly used tool for assessing life-space mobility [3]. The UAB-LSA composite score below 60 is considered to indicate restricted life-space mobility [3–5]. Empirical studies have shown that such scores correlate with reduced quality of life [6], cognitive decline [7], higher total healthcare costs and greater odds of hospitalization [8], and mortality [9]. Restricted life-space mobility may also have a negative impact on older adults’ ability to perform activities of daily living (ADLs), as our previous research has shown that a composite score of ≤ 52.3 points predicts new disability in ADLs [10]. Another study has also demonstrated that a composite score of ≤ 56 points predicts future limitations in instrumental ADLs [11].

While life-space mobility assesses actual mobility, autonomy in participation indicates the perceived possibilities to go where and when one wants to [12]. For older people, perceived limitations in participation are highest for outdoor mobility [13] and often coincide with reduced life-space mobility [14]. During the first wave of the COVID-19 pandemic, older adults experienced a decrease in life-space mobility, and they were less satisfied with their possibilities to participate in out-of-home activities compared to two years before the pandemic [15]. Although some information is available on the short-term effects of the pandemic, the longer-term implications for life-space mobility and autonomy in participation outdoors remain unclear.

Previous literature has reported extensively on the associations between life-space mobility and autonomy in participation with physical performance, especially concerning the associations with lower extremity performance, and driving [16–20]. In older age, better health, higher physical performance, and driving often coincide, and driving enables older adults with physical limitations to continue participating in activities outside the home [21, 22]. While the associations between physical performance and driving with the community mobility of older adults are well-known, their effect on changes in life-space mobility and autonomy in participation outdoors during an exceptional situation, such as the COVID-19 pandemic, has not been previously investigated.

The aim of this study was to investigate changes in community-dwelling older adults’ life-space mobility and autonomy in participation outdoors during the first wave of the COVID-19 pandemic and two years after its onset. Additionally, the aim was to study the combined effect of lower extremity performance and driving on these changes and their association with the risk of developing restricted life-space mobility. Restricted life-space mobility was determined by a threshold value of ≤ 52.3 points predicting an increased risk of ADL disability in the future [10].

## Methods

### Study design and participants

This study forms part of the ‘Active aging - resilience and external support as modifiers of the disablement outcome’ (AGNES) cohort study. Briefly, the AGNES study consists of a population-based sample with three age cohorts of people (75, 80, or 85 years old in 2017–2018) living independently in the city of Jyväskylä [23]. Jyväskylä is the seventh largest city in Finland with a population of approximately 145,890 inhabitants [24]. In addition to the city center and surrounding residential areas, Jyväskylä also includes rural areas. However, most of the participants lived in an urban environment, and at baseline, the mean population density within 1 km of the participants’ homes was 1864 inhabitants/km^2^ and the median was 1332 inhabitants/km^2^ (the source of population density [25]).

Data were collected at three time points: in a structured face-to-face home interview between September 2017 and December 2018 (baseline), in a postal survey between May and June 2020 (COVID-19 survey), and in a structured face-to-face home interview between October 2021 and October 2022 (second follow-up). The initial baseline sample consisted of 1,021 participants. The baseline study protocol, recruitment process [23, 26], and participation in the COVID-19 survey have been reported in detail previously [27].

**Fig. 1:**
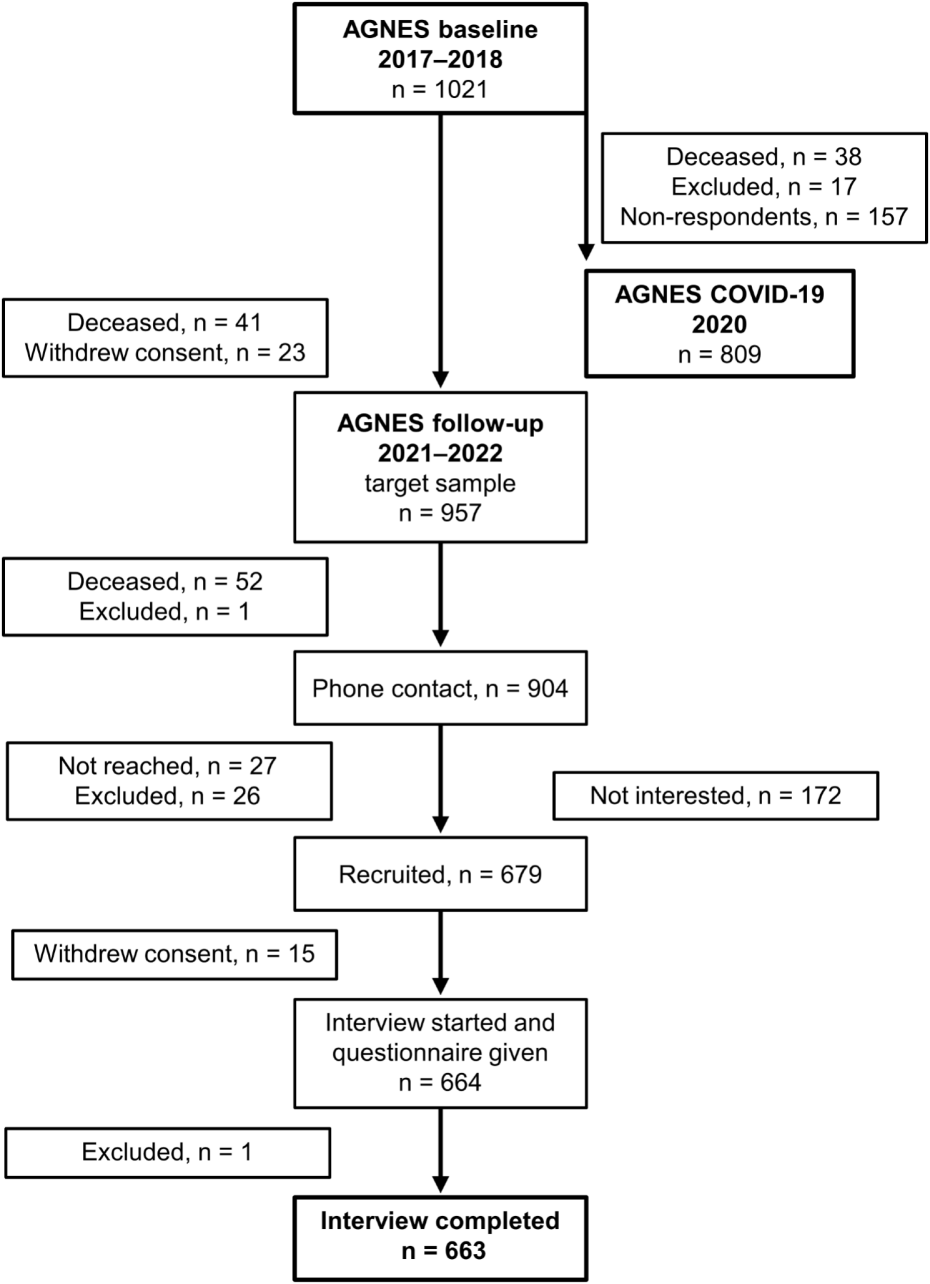
The flow chart of the study

The flow chart of the second follow-up study is shown in Fig. 1. During the first wave of the COVID-19 pandemic, the postal survey was sent to the 985 surviving baseline participants. A total of 809 people (82.1% of the target sample) participated in the COVID-19 survey. Of the 1,021 baseline participants who had not withdrawn consent, 904 survived and were contacted by telephone during the second follow-up in 2021–2022. A total of 679 participants were recruited. Fifteen participants withdrew their consent before the home interview, and one participant was excluded during the home interview due to communication difficulties. Based on the expected attrition and mortality, we expected 70–75% of the baseline participants to attend the follow-up assessments. Finally, the home interview was conducted with 663 participants, representing 73.3% of the target sample and 64.9% of the original sample.

### The COVID-19 context in Finland

The COVID-19 survey took place during the Emergency Powers Act (EPA), which was in force in Finland from 16 March to 16 June 2020. During this period, all social, cultural, and community activities were suspended. Public gatherings were limited to ten people, but there were no curfews. Extra caution was recommended for people over 70 years [28]. At the end of our data collection on 1 July 2020, there were an average of 132 cases and six deaths per 100,000 inhabitants in Finland [29].

At the beginning of the second follow-up data collection in October 2021, the COVID-19 pandemic in Finland had already lasted nearly 1.5 years. Although official restrictions were lifted in February 2022 [30], it is likely that high infection rates still influenced people’s behaviour. At the end of the second follow-up data collection on 31 October 2022, Finland had approximately 24,650 cases and 125 deaths per 100,000 inhabitants [31]. It is important to bear in mind that differences in the course of the pandemic, policies to reduce spread and transmission, and testing and reporting practices make it difficult to compare the COVID-19 context between countries. Taking this into account, the COVID-19 mortality rates in Finland, as in other northern European countries, have been among the lowest [32].

### Main variables

Life-space mobility and perceived autonomy in participation outdoors were assessed at all three time points. Life-space mobility was assessed using the University of Alabama at Birmingham Study of Aging Life-Space Assessment (UAB-LSA) [3]. The UAB-LSA is based on a self-report measure of six life-space domains, frequency of mobility, and use of assistance within the past four weeks. The composite score (range 0-120, higher scores indicating greater life-space mobility) has been suggested to be most useful for longitudinal studies [3] and was calculated for each time point. The validity and test-retest reliability of the UAB-LSA have been established in previous studies [3, 33].

Autonomy in participation outdoors was assessed using the Impact on Participation and Autonomy (IPA) subscale, which assesses a person’s self-reported satisfaction with opportunities to go to places outside the home: visit relatives and friends, go on trips and travel, spend leisure time, meet other people, and live life as one wants. Each item is scored from zero (very good) to four (very poor) [12]. The sum score ranges from zero to 20, with higher scores indicating less autonomy. The validity and test-retest reliability of the IPA has been previously established [34].

Lower extremity performance was assessed at baseline in the participants’ homes using the Short Physical Performance Battery (SPPB) with established cut-off points for scoring [35]. It has been suggested that a total score of less than ten predicts, for example, future disability and higher mortality [36]. Therefore, participants’ total score was categorized as < 10 points ‘impaired lower extremity performance’ and ≥ 10 points ‘intact lower extremity performance’.

Driving status was based on participant self-report at baseline. Participants were asked: *“How often do you drive a car by yourself?”* and responses were categorised as drivers (at least a few times per week) and non-drivers (a few times per month or less).

### Covariates

Age and sex were obtained from the Digital and Population Data Services Agency in the context of sampling. Self-reported total number of years of education and perceived economic situation (very good/good or fair/poor) were used to indicate socioeconomic status. The total number of chronic diseases was calculated from a list of self-reported physician-diagnosed chronic conditions. Depressive symptoms were assessed using the 20-item Centre for Epidemiologic Studies Depression Scale (CES-D; range 0–60, with higher scores indicating more depressive symptoms) [37]. Cognitive function was assessed using the Mini-Mental State Examination (MMSE; range 0–30, with higher scores indicating better cognitive function) [38].

### Statistical analyses

The final analysis comprised 657 participants with outcome variables from at least two time points. At baseline, 12 participants lacked information on driving. For these participants, information was imputed based on a later questionnaire. As both lower extremity performance and driving status were expected to correlate with life-space mobility, we categorised participants into four groups to assess their combined effects: *SPPB ≥ 10 and driving*, *SPPB ≥ 10 and not driving*, *SPPB < 10 and driving*, and *SPPB < 10 and not driving*.

Participant characteristics are reported as means (M) and standard deviations (SD) for continuous variables and as percentages (%) for nominal variables. Differences in background characteristics between responders and non-responders and between SPPB and driving status categories were tested using the chi-squared test for categorical variables and the independent samples t-test or one-way analysis of variance (ANOVA) for continuous variables.

Generalized estimating equations (GEE) [39] with a linear link function and unstructured working correlation matrix were used to determine whether life-space mobility and autonomy in participation outdoors (group effect) and their change over time (group by time interaction) differed between SPPB and driving status categories. GEE models were adjusted for age, sex, years of education, perceived economic situation, number of chronic conditions, MMSE, and CES-D. Regression coefficients (B), standard errors (SE), and p-values are reported. In the case of missing data for the outcome variables, multivariate imputation by chained equations (MICE) was used to calculate the missing total score for life-space mobility (2020: *n* = 56, 2021-22: *n* = 3) and for autonomy in participation outdoors (2017-18: *n* = 6, 2020: *n* = 66, 2021-22: *n* = 6). Excluding participants with the imputed parameters did not change the results remarkably based on sensitivity analysis (data not shown).

The UAB-LSA composite score ≤ 52.3 was used to indicate restricted life-space mobility [10]. Cox proportional hazards regression was used to analyse the risk of developing restricted life-space mobility among those above the cut-off at baseline (*n* = 567). In these analyses, SPPB and driving status were analysed first separately and then using their combined distributions. Ties were handled using the Breslow method. In the analyses, participants were censored at the time they reported restricted life-space mobility or at the end of the follow-up, whichever came first. Results are presented as relative risks (RR) with 95% confidence intervals (CI). Model 1 is unadjusted. In model 2, age, sex, and years of education were added, and in model 3, perceived economic situation, number of chronic conditions, MMSE, and CES-D were also added. A model including only SPPB was adjusted for driving status and vice versa. The significance level was set at 0.05, and all the analyses were performed using the SPSS statistical software package (IBM SPSS Statistics Version 28.0.1.1).

## Results

The mean follow-up time was 3.9 (SD 0.3, range 3.0-5.2 years) years from baseline to the second follow-up. The participation rate at the second follow-up was 64.9% and did not differ between the sexes (*p* = 0.679). Follow-up responders were younger, drove more often, had more years of education, better physical performance, better cognitive function, fewer chronic diseases, and fewer depressive symptoms at baseline than non-responders (*p* < 0.05 for all) (Supplementary Table S1). Baseline characteristics of participants categorised according to SPPB and driving status are shown in Table 1.

**Table 1:** Baseline characteristics of participants by SPPB total score and driving status category (*n* = 657)

Characteristics	1 SPPB ≥ 10, driving *n* = 324	2 SPPB ≥ 10, not driving *n* = 174	3 SPPB < 10, driving *n* = 61	4 SPPB < 10, not driving *n* = 98	p-value
	%	%	%	%	
Sex (female)	34.9	89.0	42.6	86.7	< 0.001 ^1: *acdf*^
Age cohort					< 0.001 ^1: *abc*^
75 years	56.2	46.0	34.4	41.8	
80 years	34.9	36.2	39.3	30.6	
85 years	9.0	17.8	26.2	27.6	
Perceived economic situation					< 0.001 ^1: *c*^
Very good / good	69.7	58.2	54.1	50.0	
Fair / poor	30.3	41.8	45.9	50.0	
	Mean (SD)	Mean (SD)	Mean (SD)	Mean (SD)	
Total years of education	12.5 (4.5)	11.2 (4.2)	11.7 (4.3)	10.5 (3.5)	< 0.001 ^2: *ac*^
Number of chronic diseases	2.8 (1.7)	3.1 (1.9)	3.7 (1.8)	4.1 (2.1)	< 0.001 ^2: *bce*^
CES-D score (range 0–60)	6.5 (5.5)	7.9 (6.7)	11.1 (9.4)	11.1 (8.4)	< 0.001 ^2: *bce*^
MMSE score (range 0–30)	27.9 (1.8)	27.6 (2.2)	27.3 (2.2)	26.9 (2.5)	< 0.001 ^2: *c*^

SD = Standard Deviation; SPPB = Short Physical Performance Battery; CES-D = Center for Epidemiologic Studies Depression Scale; MMSE = Mini-Mental State Examination

^1^ Tested with Chi-Square test. ^2^ Tested with one-way analyses of variance. Bonferroni-corrected pairwise comparison (*p* < 0.05): a = 1 vs. 2; b = 1 vs. 3; c = 1 vs. 4; d = 2 vs. 3; e = 2 vs. 4; f = 3 vs. 4

**Fig. 2:**
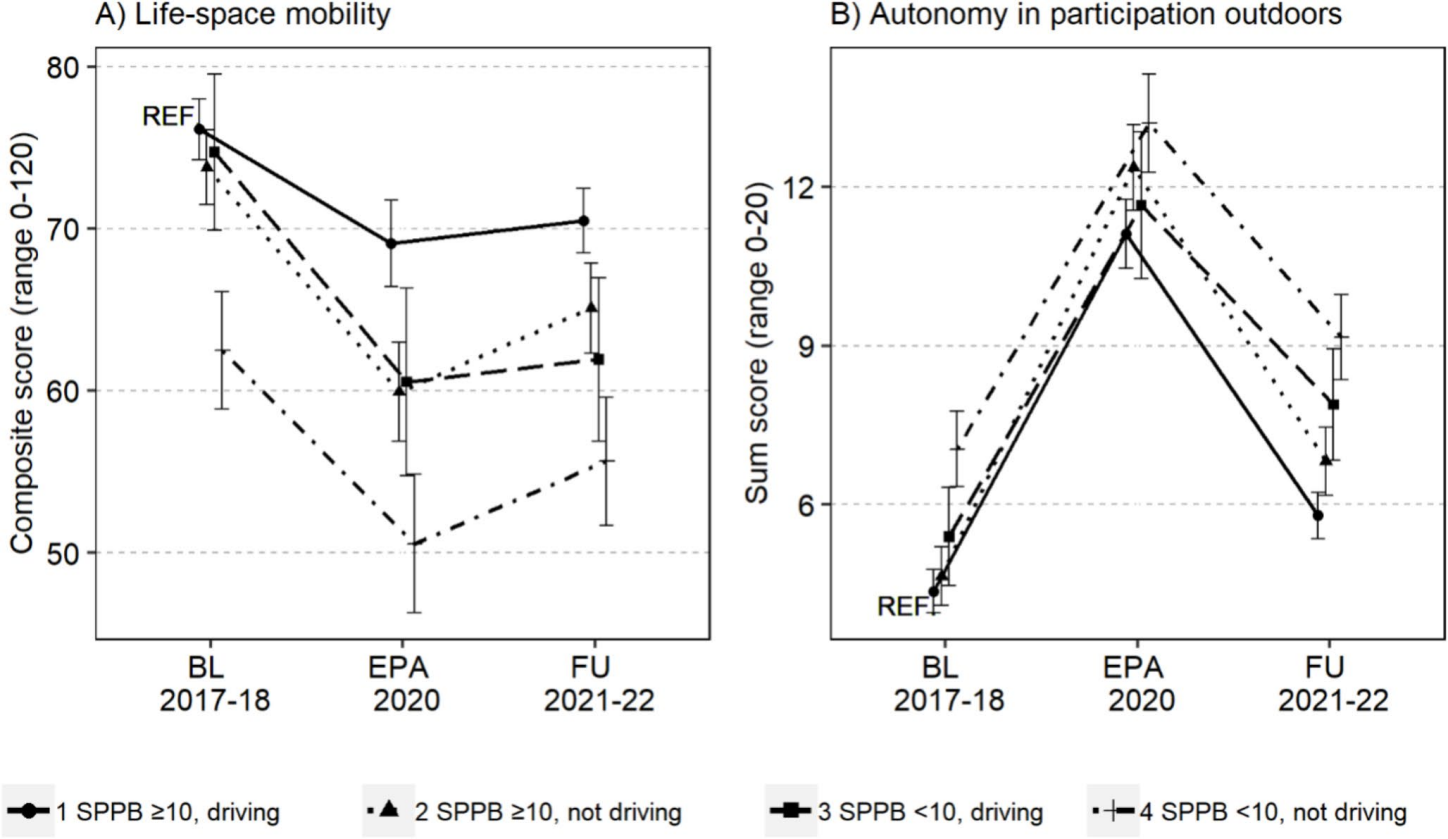
**(A)** Life-space mobility (higher scores indicate greater life-space mobility) and **(B)** autonomy in participation outdoors (higher scores indicate weaker autonomy) at baseline (BL) in 2017–2018, during the Emergency Powers Act (EPA) in 2020, and at the second follow-up (FU) in 2021–2022 according to the SPPB total score and driving status categories. Data are presented as estimated marginal means (adjusted for sex, age, years of education, perceived economic situation, cognitive function, depressive symptoms, and number of chronic diseases) with 95% confidence intervals. SPPB ≥ 10 and driving are used as a reference group (REF)

During the Emergency Powers Act (EPA) in 2020, the average reduction in life-space mobility was 10.3 (SD 21.6) points compared to baseline (M 74.2, SD 17.9 vs. M 63.9, SD 24.0, *p* < 0.001). Some increase was observed at the second follow-up in 2021–2022, with a mean recovery of 2.7 (SD 21.8) points (M 66.6, SD 20.3, *p* < 0.01). Changes over time in life-space mobility between SPPB and driving status categories are shown in Table 2; Fig. 2A. From baseline to the EPA in 2020, life-space mobility decreased more sharply in other SPPB and driving status categories than in the reference group, i.e., drivers with intact lower extremity performance (*p* < 0.05). The rate of change from EPA to the second follow-up was not statistically significant between the categories. As life-space mobility declines with age, additional age cohort comparisons were performed, which showed that the trajectories of life-space mobility from baseline to EPA or from EPA to the second follow-up in 2021–2022 did not differ between the initial age groups (*p* = 0.619 and *p* = 0.087, respectively), even though the starting values differed.

**Table 2:** Changes in life-space mobility composite scores and autonomy in participation outdoors sum scores from pre-COVID-19 to pandemic over the follow-up period by SPPB total score and driving status category (*n* = 657)

	Group effect			BL to EPA Group ^x^ Time			EPA to FU Group ^x^ Time			BL to FU Group ^x^ Time	
	B (SE)	p		B (SE)	p		B (SE)	p		B (SE)	p
**Life-space moblity**											
SPPB ≥ 10, driving	Ref.										
SPPB ≥ 10, not driving	-2.3 (1.5)	0.117		-6.8 (2.1)	< 0.001		3.8 (2.1)	0.074		-3.0 (1.6)	0.062
SPPB < 10, driving	-1.4 (2.6)	0.589		-7.1 (3.3)	0.030		-0.01 (3.0)	0.996		-7.1 (2.9)	0.014
SPPB < 10, not driving	-13.6 (2.1)	0.036		-4.9 (2.3)	0.036		3.7 (2.1)	0.137		-1.2 (2.0)	0.543
**Autonomy in participation outdoors**											
SPPB ≥ 10, driving	Ref.										
SPPB ≥ 10, not driving	0.3 (0.3)	0.410		1.0 (0.5)	0.067		-0.2 (0.5)	0.674		0.7 (0.3)	0.019
SPPB < 10, driving	1.0 (0.5)	0.043		-0.5 (0.9)	0.577		1.6 (0.8)	0.060		1.1 (0.5)	0.035
SPPB < 10, not driving	2.7 (0.4)	< 0.001		-0.6 (0.6)	0.305		1.3 (0.6)	0.027		0.7 (0.4)	0.108

B = the population-averaged coefficient, SE = standard error, BL = baseline in 2017–2018, EPA = Emergency Powers Act in 2020, FU = second follow-up in 2021–2022, SPPB = Short Physical Performance Battery. All GEE models are adjusted for sex, age cohort, years of education, perceived financial situation, number of chronic diseases, cognitive function and depressive symptoms

Perceived restrictions in autonomy in participation outdoors increased by a mean of 6.9 (SD 5.4) points from baseline to the EPA (M 4.9, SD 3.7 vs. M 11.7, SD 5.2, *p* < 0.001) and recovered by a mean of 5.0 (SD 5.5) points by the second follow-up (M 6.7, SD 4.3, *p* < 0.001). Changes over time in perceived autonomy in participation outdoors between SPPB and driving status categories are shown in Table 2; Fig. 2B. The rate of change in perceived autonomy in participation outdoors was not statistically significant between categories from baseline to the EPA. However, from the EPA to the second follow-up, the slope of recovery was more modest among non-drivers with impaired lower extremity performance compared to the reference group (*p* = 0.027).

Table 3 shows that older adults with poor lower extremity performance, either alone or combined with non-driver status, were at a higher risk of restricted life-space mobility. Non-drivers with impaired lower extremity performance were 3.6 times (95% CI 1.8–7.4) more likely, drivers with impaired lower extremity performance were 2.5 times (95% CI 1.2–5.1) more likely, and non-drivers with intact lower extremity performance were 2.4 times (95% CI 1.2–4.5) more likely to develop restricted life-space mobility than drivers with intact lower extremity performance, even after adjusting for all covariates.

**Table 3:** Effect of SPPB and driving status on the risk of restricted life-space mobility over a four-year follow-up period in community-dwelling older adults without restricted life-space mobility at baseline (*n* = 567)

		The proportion of restricted life-space mobility in the second follow-up in 2021-22	Risk of developing restricted life-space mobility					
	**n**	**%**	Model 1		Model 2		Model 3	
			RR (95% CI)		RR (95% CI)		RR (95% CI)	
**Single effect**								
SPPB ≥ 10 - Ref.	461	14.1	1.0		1.0		1.0	
SPPB < 10	106	34.9	**2.6 (1.7, 3.9)**		**2.1 (1.4, 3.2)**		**1.8 (1.2, 2.8)** ^**A**^	
Driving - Ref.	354	10.7	1.0		1.0		1.0	
Not driving	213	30.0	**2.8 (1.9, 4.3)**		**2.1 (1.2, 3.5)**		**2.0 (1.1, 3.4)** ^**B**^	
**Combined effect**								
SPPB ≥ 10, driving - Ref.	303	7.6	1.0		1.0		1.0	
SPPB ≥ 10, not driving	158	26.6	**3.5 (2.1, 5.9)**		**2.7 (1.4, 4.9)**		**2.4 (1.2, 4.5)**	
SPPB < 10, driving	51	29.4	**4.1 (2.1, 7.8)**		**3.3 (1.7, 6.5)**		**2.5 (1.2, 5.1)**	
SPPB < 10, not driving	55	40.0	**5.5 (3.0, 9.8)**		**3.8 (1.9, 7.5)**		**3.6 (1.8, 7.4)**	

RR = relative risk; 95% CI = 95% confidence interval; SPPB = Short Physical Performance Battery. Cox regression: Model 1 unadjusted; Model 2 adjusted for sex, age, and years of education; Model 3 adjusted for sex, age, years of education, perceived economic situation, cognitive function, depressive symptoms, number of chronic diseases, and ^A^ driving status or ^B^ SPPB categories. Statistically significant values are in bold

## Discussion

This four-year prospective study found that the reductions in life-space mobility and autonomy in participation outdoors, observed in older adults during the Emergency Powers Act (EPA) in 2020, were not entirely reversed during the second follow-up in 2021–2022. The findings also indicated that impaired lower extremity performance and non-driving status were associated with an increased risk of falling below the threshold of restricted life-space mobility, which predicts an increased risk for ADL disability in the future. To our knowledge, this study is the first to report on the impact of the prolonged pandemic on older adults’ life-space mobility and autonomy in participation outdoors.

Under normal conditions, the expected decline in life-space mobility in the current age group within 1–2 years is approximately 2–4 points [6, 10], and less than a 1-point increase in the score of perceived autonomy in participation outdoors [40]. A change of 5 to 10 points in life-space mobility is clinically meaningful [3–5]. In terms of autonomy in participation outdoors, a meaningful change is still unknown, but it can be argued that any change could be meaningful because it reflects people’s immediate perceptions of their possibilities to live as they wish [40]. As the changes observed in this study were greater than those mentioned above, it is reasonable to argue that the COVID-19 pandemic caused a meaningful change, at least at the beginning of the pandemic, by accelerating the decline in life-space mobility and autonomy in participation outdoors. However, distinguishing between the effects of the pandemic and ageing becomes more difficult in the second follow-up, as, for example, life-space mobility has been reported to decline with age [41].

Our findings suggest that the combined effect of two key factors that positively influence older adults’ community mobility, namely lower extremity performance and driving, became more pronounced during the COVID-19 pandemic. Good physical performance provides older adults independence in daily tasks and reduces dependence on others [42]. This was highlighted during the pandemic when interactions with people outside the household were limited. Driving, which is often associated with greater independence in mobility [43], also provided a safer travel option during the pandemic than public transport, which instilled fear due to the potential risk of virus exposure [44]. The benefits of having good lower extremity performance and driving a car were more apparent in life-space mobility, which measures actual mobility. It appears that the pandemic affected everyone’s perceived opportunities to participate in activities outside the home, which may explain the smaller effects observed in perceived autonomy in participation outdoors.

According to our results, non-drivers with impaired lower extremity performance were the most likely to develop restricted life-space mobility during the follow-up. Consequently, they face the highest risk of future ADL decline, especially considering their compromised health and physical performance at baseline. Given that both impaired lower extremity performance [45] and non-driving [46] are more prevalent among women, it is likely that women faced a higher risk of reduced life-space mobility during the pandemic. Our results suggest that the impact of the COVID-19 pandemic on older people’s mobility varied according to their resources. The most vulnerable community-dwelling older adults may need targeted attention and support to maintain and restore their community mobility.

This study has several strengths, including a relatively large population-based sample with longitudinal data, and well-established self-rated and performance-based measures [3, 33–36]. However, there are some limitations to consider, such as the inability to fully separate the effect of the COVID-19 pandemic from ageing and declining health. When generalizing the results, it should be noted that Finland’s low population density and COVID-19 restrictions without any curfew may limit the generalizability of our results to countries with higher population densities or stricter restrictions. Regarding the target group, it is worth mentioning that non-responders were older and in poorer health, and the minority of non-drivers were men. Moreover, non-drivers included people with a variety of transport options (e.g., passengers in private cars or passengers on public transport). To account for seasonal variations, both the baseline and the second follow-up were conducted during the same season. However, the 2020 survey was carried out in early summer, which could have influenced the results for those whose other measurements were taken in winter. Additionally, the varying government strategies to control the pandemic may have affected the results of the participants at the second follow-up.

## Conclusions

The COVID-19 pandemic accelerated the decline in life-space mobility and reduced the autonomy in participation outdoors. Older adults with limited resources for community mobility, i.e. with impaired lower extremity performance and who did not drive, were more likely to develop reduced life-space mobility during the follow-up period, potentially leading to a vicious cycle of social isolation, low physical activity, and further disability.

### Electronic supplementary material

Below is the link to the electronic supplementary material.

Supplementary Material 1

## Data Availability

The authors confirm that some access restrictions apply to the data. Researchers interested using the data must obtain approval from the director of the AGNES study, Professor Taina Rantanen (taina.rantanen@jyu.fi), and are required to follow the protocol on the protection of privacy and to comply with the relevant Finnish laws.
