# Supplementary material for: Longitudinal changes in life-space mobility and autonomy in participation outdoors among Finnish community-dwelling older adults from pre-COVID-19 to through the pandemic
Source: Aging Clin Exp Res. 2024 Apr 1;36(1):85. doi: 10.1007/s40520-024-02734-6 (PMC10984880; doi:10.1007/s40520-024-02734-6)
Supplement: Supplementary file 1 — Supplementary Material 1 [file 40520_2024_2734_MOESM1_ESM.docx]

**Supplementary material**

**Table S1.** Comparison of baseline characteristics between the second follow-up responders and non-responders

|  | **Responders (n = 663)** | **Non-responders (n = 358)** | **p** |
| --- | --- | --- | --- |
|  | **%** | **%** |  |
| Sex (female) | 57.8 | 56.4 | .679 ^a^ |
| Initial age cohort |  |  | **<.001 ^a^** |
| 75 years | 49.0 | 37.2 |  |
| 80 years | 35.0 | 29.1 |  |
| 85 years | 16.0 | 33.8 |  |
| Driving a car |  |  | **<.001 ^a^** |
| Drive regularly | 58.9 | 47.3 |  |
| Perceived economic situation |  |  |  |
| Very good / good | 62.0 | 56.7 | .099 ^a^ |
|  |  |  |  |
|  | **Mean (SD)** | **Mean (SD)** |  |
| Years of education | 11.8 (4.3) | 10.9 (4.0) | **<.001 ^b^** |
| Number of chronic conditions | 3.3 (2.0) | 3.6 (2.1) | **.017 ^b^** |
| SPPB score (range 0-12) | 10.3 (1.9) | 9.0 (2.9) | **<.001 ^b^** |
| MMSE score (range 0-30) | 27.6 (2.1) | 26.3 (3.1) | **<.001 ^b^** |
| CES-D score (range 0-60) | 7.9 (7.0) | 10.0 (7.2) | **<.001 ^b^** |

Note: SPPB = Short Physical Performance Battery; MMSE = Mini-Mental State Examination; CES-D = Center for Epidemiologic Studies Depression Scale,

^a^ Tested with Chi-Square test. ^b^ Tested with independent Sample T-test.
